# Neurobehavioral Abnormalities in the HIV-1 Transgenic Rat Do Not Correspond to Neuronal Hypometabolism on ^18^F-FDG-PET

**DOI:** 10.1371/journal.pone.0152265

**Published:** 2016-03-24

**Authors:** William C. Reid, Rafael Casas, Georgios Z. Papadakis, Siva Muthusamy, Dianne E. Lee, Wael G. Ibrahim, Anand Nair, Deloris Koziol, Dragan Maric, Dima A. Hammoud

**Affiliations:** 1 Center for Infectious Disease Imaging (CIDI), Radiology and Imaging Sciences, National Institutes of Health, Bethesda, Maryland, United States of America; 2 Biostatistics and Clinical Epidemiology Service, Clinical Center, National Institutes of Health, Bethesda, Maryland, United States of America; 3 Division of Intermural Research (DIR), National Institute of Neurological Disorders and Stroke (NINDS), National Institutes of Health, Bethesda, Maryland, United States of America; University of Nebraska Medical Center, UNITED STATES

## Abstract

Motor and behavioral abnormalities are common presentations among individuals with HIV-1 associated neurocognitive disorders (HAND). We investigated whether longitudinal motor and behavioral performance in the HIV-1 transgenic rat (Tg), a commonly used neuro-HIV model, corresponded to *in vivo* neuronal death/dysfunction, by using rotarod and open field testing in parallel to [^18^F] 2-fluoro-2-deoxy-D-glucose (FDG) positron emission tomography (PET). We demonstrated that age-matched non-Tg wild type (WT) rats outperformed the HIV-1 Tg rats at most time points on rotarod testing. Habituation to rotarod occurred at 8 weeks of age (fifth weekly testing session) in the WT rats but it never occurred in the Tg rats, suggesting deficits in motor learning. Similarly, in open field testing, WT rats outperformed the Tg rats at most time points, suggesting defective exploratory/motor behavior and increased emotionality in the Tg rat. Despite the neurobehavioral abnormalities, there were no concomitant deficits in ^18^F-FDG uptake in Tg rats on PET compared to age-matched WT rats and no significant longitudinal loss of FDG uptake in either group. The negative PET findings were confirmed using ^14^C- Deoxy-D-glucose autoradiography in 32 week-old Tg and WT rats. We believe that the neuropathology in the HIV-1 Tg rat is more likely a consequence of neuronal dysfunction rather than overt neurodegeneration/neuronal cell death, similar to what is seen in HIV-positive patients in the post-ART era.

## Introduction

Prior to the advent of highly active antiretroviral therapy (HAART), HIV associated cognitive impairment, initially referred to as AIDS dementia complex (ADC), was a frequent complication as subjects progressed to acquired immunodeficiency syndrome (AIDS). HAART therapy has resulted in markedly decreased incidence of ADC. However, reports have described an increase in the development of milder forms of cognitive impairment, part of the spectrum of HIV-1 associated neurocognitive disorders (HAND), affecting 15–50% of infected individuals[1–3]. Despite the gravity of the HIV associated cognitive impairment, there are still no accurate preclinical *in vivo* biomarkers of brain pathology or biomarkers in patients to gauge responses to preventative therapy [4].

The HIV-1 transgenic (Tg) rat is used by many groups as a small animal model of neuro-HIV [5–14]. The Tg rat expresses 7 of the 9 HIV-1 viral proteins including gp120, nef, and tat and is known to develop clinically relevant neuro-pathologies and cognitive deficits [11, 15–18]. Motor and behavioral abnormalities have been previously reported in the Tg rat [6, 8, 10, 12, 15, 19–22]. The aim of our study was to investigate whether dysregulated motor and anxiety-like behavioral changes corresponded to *in vivo* neuronal integrity/survival, which was measured using [18F] 2-fluoro-2-deoxy-D-glucose (^18^F-FDG) and positron emission tomography (PET). Motor and behavioral assessments were determined by rotarod and open field testing in parallel to ^18^F-FDG-PET imaging. Additionally, at the end of the study, ^18^F-FDG-PET uptake was confirmed by *ex vivo* autoradiography, performed on selected brain sections, using 2-[1-^14^C] Deoxy-D-glucose (^14^C-DG) uptake.

## Materials and Methods

### Animals

Male HIV-1 Tg (F344/Hsd, Tg) and age-matched wild type (F344/Hsd, WT) rats were purchased from Harlan Inc. (Indianapolis, IN) and used in various experiments. All rats were housed in a temperature-controlled environment with a 12-hour light/dark cycle with free access to food and water; procedures were conducted during the light cycle. All studies were approved by the Animal Care and Use Committee of the National Institutes of Health/Clinical Center.

### Motor Function

#### Rotarod

Data on changes in motor function and habituation rate (non-adaptive learning) from Tg and WT rats were collected using a single station Rotarod for rats (Med Associates, Inc. St. Albans, VT). A total of 9 rats in two groups (4 WT and 5 Tg rats) were tested starting at 4 weeks of age, and then every 1–2 weeks over a period of twenty weeks for a total of thirteen time points. Each time point consisted of 2 trials, which were averaged.

One week prior to testing, animals received three days of training, during which acclimation to the room, apparatus and experimenter occurred. Even though we did not find observable differences in performance during the training period between morning and afternoon testing, all subsequent trials were conducted at the same time of the day (10:00 AM-12:00 PM), which also corresponded to the timing of the PET studies. On testing days, rats were transported together and acclimated to the testing room for 15 minutes. The order of testing was randomized for each trial; the apparatus was cleaned between tests with 70% ethanol to remove olfactory clues of human or rat origin. Each rat was removed from its cage and was placed on the rod in the direction of rotation. The rotarod has a default speed of 4 revolutions per minute (rpm) which accelerates to 40 rpm over the 5 minute testing period. Data was collected as the latency to fall which is detected by a photo-beam under the rotating shaft of the apparatus.

Habituation was assumed to occur at the time point when significant differences in performance (of either group) were reached compared to the corresponding baseline.

### Behavioral Assessment

#### Open Field

The same groups of animals used in the longitudinal assessment of rotarod performance (4 WT and 5 Tg rats) were also tested for open field activity. Testing began when subjects were 11 weeks of age and seven time points were collected over the following 14 weeks.

The open field apparatus has a square floor with side lengths of 40 cm, surrounded by 35 cm high opaque Plexiglas walls, an overhead recording camera, photo-beam array (to detect rearing behavior) and tracking software (used for tracking and analyzing data in pre-defined zones). Each animal was subjected to a 10 minute preconditioning period one-week prior to data collection; during this time, acclimation to the room, apparatus and experimenter occurred. On testing days, rats were transported together and acclimated to the testing room for 30 minutes. The order of testing was randomized for each experimental trial and the apparatus was cleaned with 70% ethanol between tests. All trials were conducted at the same time of the day (10:00AM-4:00 PM) for 30 minute intervals. Under low illumination, tracking was initiated once the animal was placed in the center of the field. Measures of total exploratory activity, distance traveled (interior zone), distance travelled (external zone), number of line crossings, rearing time, and rearing time (external zone) were collected for analysis.

### Radioligand Preparation

The radiosynthesis of ^18^F-FDG was performed by Cardinal Health Pharmacy (Beltsville, MD) and delivered on the days of scanning to the NIH Clinical Center Micro-PET facility. ^14^C-DG, in sterile saline, (specific activity: 55 mCi/mmol; concentration: 0.125 mCi/ml) was purchased from American Radiolabeled Chemicals Inc. (St Louis, MO, USA).

### ^18^F-FDG PET Scanning

For the longitudinal study, 5 male Tg and 4 aged-matched WT rats were scanned starting at four weeks of age, and then approximately every four weeks until 31-weeks of age. In a separate cross-sectional study, we performed PET imaging on 5 young Tg and 5 age-matched WT rats (mean age = 12.1 (0.9) and 12.1 (0.8) weeks respectively) as well as 5 middle-aged Tg and 4 age-matched WT rats (mean age = 29 (0.1) and 29.7 (0.5) weeks respectively). For both the longitudinal and cross-sectional study animals, the order of scanning was always counterbalanced: one Tg and one WT, in alternating order; four rodents were scanned per day. Imaging was always preformed within at least three days of any behavioral testing in order to avoid the potential effect of exercise on FDG uptake [23–25]. Rats were weighed and placed individually in an induction chamber containing oxygen (O_2_)—anesthetic gas mixture (O_2_ 3–4 L/min and Isoflurane at 4%). Rats were then removed from the induction chamber and maintained in a deep plane of anesthesia by using a nose-cone adapter and adjusting the O_2_ flow rate to 2-3L/min and Isoflurane to 1.5–3.0%. Each rat subsequently received an intravenous (IV) injection of ^18^F-FDG (0.7–1.2 mCi) via the tail vein. Animals were allowed to regain consciousness and after an uptake period of 30 minutes, were again anesthetized (as described above), and positioned into the Siemens Inveon Multimodality scanner (Siemens Medical Solutions USA, Inc.) in a head-first-prone position, on a heating pad maintained at 37°C. A CT scan was performed for localization and attenuation correction purposes. All CT data were acquired with a voltage set to 60 keV, a tube current of 500 μA and a total rotation angle of 220°, with 120 rotation steps. The head and thorax were scanned in 3 bed positions of 57.55 mm each. Subsequently, a 20 minute static head PET scan was acquired. The scanner uses 64 lutetium oxyorthosilicate block detectors arranged in 4 contiguous rings of 16 blocks, with a 16.1 cm ring diameter allowing for a transaxial field of view (FOV) of 10 cm and an axial FOV of 12.7 cm. The spatial resolution of the scanner at the center of the transaxial and axial FOV is 1.46 mm, 1.49 mm and 1.15 mm in the radial, tangential and axial directions, respectively. PET images were reconstructed using OSEM3D/MAP algorithm, with Ramp projection filter, scattered corrected, 2 OSEM3D iterations, 18 MAP iterations, 128 × 128 image size, and approximately 0.5mm resolution at the center of the FOV. After reconstruction, individual PET images were loaded into PMOD v3.5 (PMOD Technologies, Switzerland). Each PET image was then manually registered to the same MR rat brain template. Volumes of interest (VOIs) of the Striatum, Cortex (somatosensory and motor), and Hippocampus pre-drawn on the template were used to generate the region values in kilobecquerel per cubic centimeter (kbq/cc). Standardized uptake values (SUV) were then calculated for each region by adjusting for the weight of the animal. To account for variability of SUV in small animals, these values were normalized to whole brain uptake.

### ^14^C-DG Autoradiography

Seven to fifteen days following the completion of the PET study, 7–8 month old rat groups that were in the longitudinal study (4 Tg, 4 WT) were sacrificed. Each rat was anesthetized (as described above) and 125 μCi/kg body weight of ^14^C-DG was administered by tail vein injection. Rats were then allowed to regain consciousness and following an uptake period of 45 minutes, were euthanized by Isoflurane overdose, decapitated and brains rapidly removed. These brains were briefly submerged in 0.3M sucrose on ice, placed into a brain matrix (World Precision Instruments, Sarasota, FL), cut into coronal sections (3 mm each), placed into embedding medium (O.C.T., Tissue-Tek^®^, Sakura Finetek U.S.A., Inc., Torrance, CA), frozen on dry ice and stored at −80°C. Brain blocks were cut with a cryostat (Leica Microsystems, Germany; Model CM1850-3-1) at -12 to -18°C into 20-μm thick coronal sections and dried on glass slides. Brain sections were covered with scintillator sheets (Biospace Lab; Paris, France) and imaged for 18 hours using a MicroImager (Biospace Lab; Paris, France). All samples were imaged simultaneously with the [^14^C] standards (American Radiolabeled Chemicals Inc., St Louis, MO, USA). Identical regions of interest (ROI) were selected for the cortex (0.752 mm^2^), and striatum (2.459 mm^2^). The ROI’s for the hippocampi were drawn manually to encompass their entirety and total counts were then normalized to the area. All counts obtained were standardized against the [^14^C] standards and values were converted to nCi/g of tissue.

### Statistics

Longitudinal open field and rotarod data were analyzed using mixed models for repeated measures (SAS/STAT Software 9.2, SAS Institute, Inc., Cary, NC). Separately, for the rotarod and each open field assessment outcome, the predictor variable Group (WT versus Tg) was modeled as a fixed effect and days as the repeating variable. The covariance pattern across days was structured as first-order autoregressive and the Kenward-Roger method was used for computing the degrees of freedom for tests of fixed effects. The SAS estimator was the least square mean and the measure of variability was the standard error. A p-value of ≤ 0.05 was considered significant. As a determinate of habituation for the rotarod data, (separately for WT and Tg rats), interval differences of least square means from baseline were compared using t-tests at each follow-up. These post-hoc p-values were adjusted using the step down Bonferroni method or (Holm); p≤ 0.05 were considered significant.

For ^18^F-FDG uptake values, both the longitudinal and the cross-sectional comparisons between Tg and age-matched WT rats at each time point were performed using an unpaired Student’s *t*-test for each of the 3 selected regions (Striatum, Hippocampus, and Cortex). The SUV values used were corrected for whole brain uptake. The p values were corrected for Type I errors using the Holm-Šídák approach.

For the longitudinal comparison, we performed paired Student’s *t*-test to compare the baseline SUV values (5 week-old) to the last time point (31-week-old) SUV values for the Tg and WT rats separately.

Autoradiography data were compared using the Mann-Whitney U-test for unpaired nonparametric data. Data were tested for normal distribution prior to analysis and a p-value of ≤ 0.05 was considered significant.

## Results

### Motor Function

#### Rotarod

**Fig 1:**
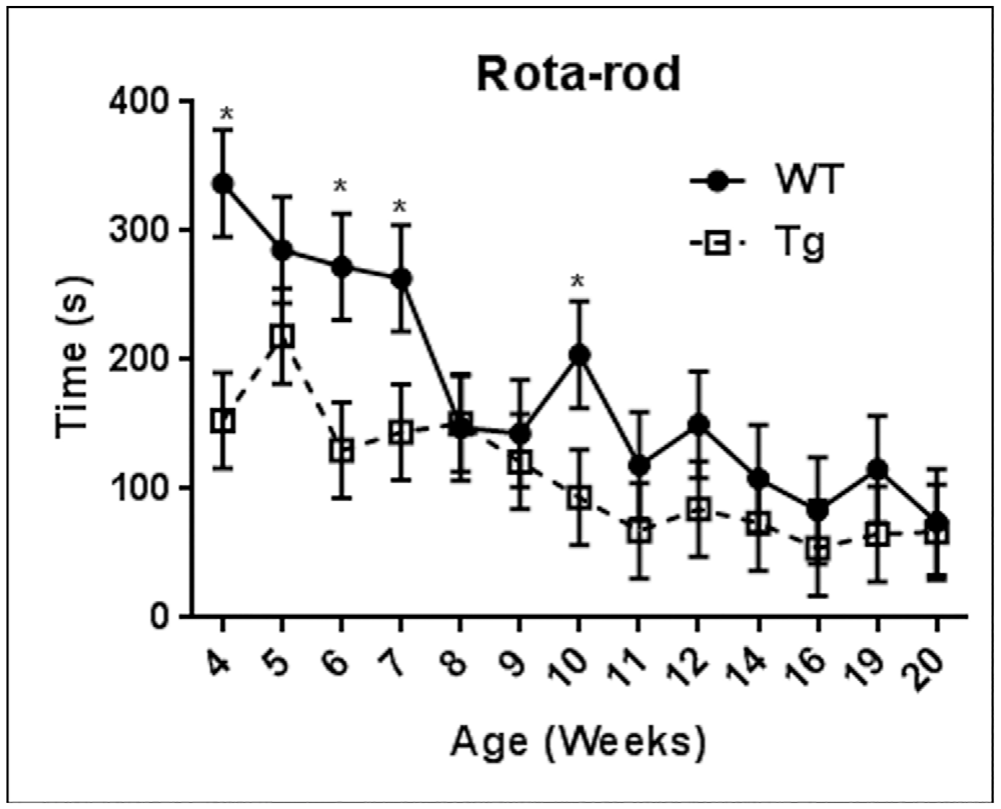
Longitudinal Rotarod performance measures of HIV-1 Tg and age-matched WT rats. In the longitudinal measure, the transgene state (group) and age were found to be significant predictors of Rotarod performance [p<0.05], with the group and age interaction not statistically significant [p>0.05]. By week 8, control rats demonstrated significantly different Rotarod times compared to baseline, whereas Tg rats did not show a significant difference at any time point. Error bars reflect standard error of the mean (SEM) values. * reflect differences between WT and Tg rats (p<0.05).

Throughout the testing period the WT control rats generally outperformed the Tg rats (Fig 1). The transgene state (group) and age were found to be significant predictors of rotarod performance, respectively [F(1,18.1) = 10.32, p = 0.0048] and [F(12,74.3) = 3.50, p = 0.0004]; however, the group and age interaction was not significant [F(12,74.3) = 1.14, p = 0.3407]. Interestingly, by the eighth week after starting the testing (fifth testing session), WT rats demonstrated significantly different rotarod performance (habituation) compared to baseline (p = 0.031). Tg rats on the other hand never showed a significant difference from baseline (Fig 1). In four out of the thirteen time points, the WT rats significantly outperformed the Tg rats. Those differences became non-significant after ten weeks of age, presumable because of WT habituation.

### Behavior Assessment

#### Open field

**Fig 2:**
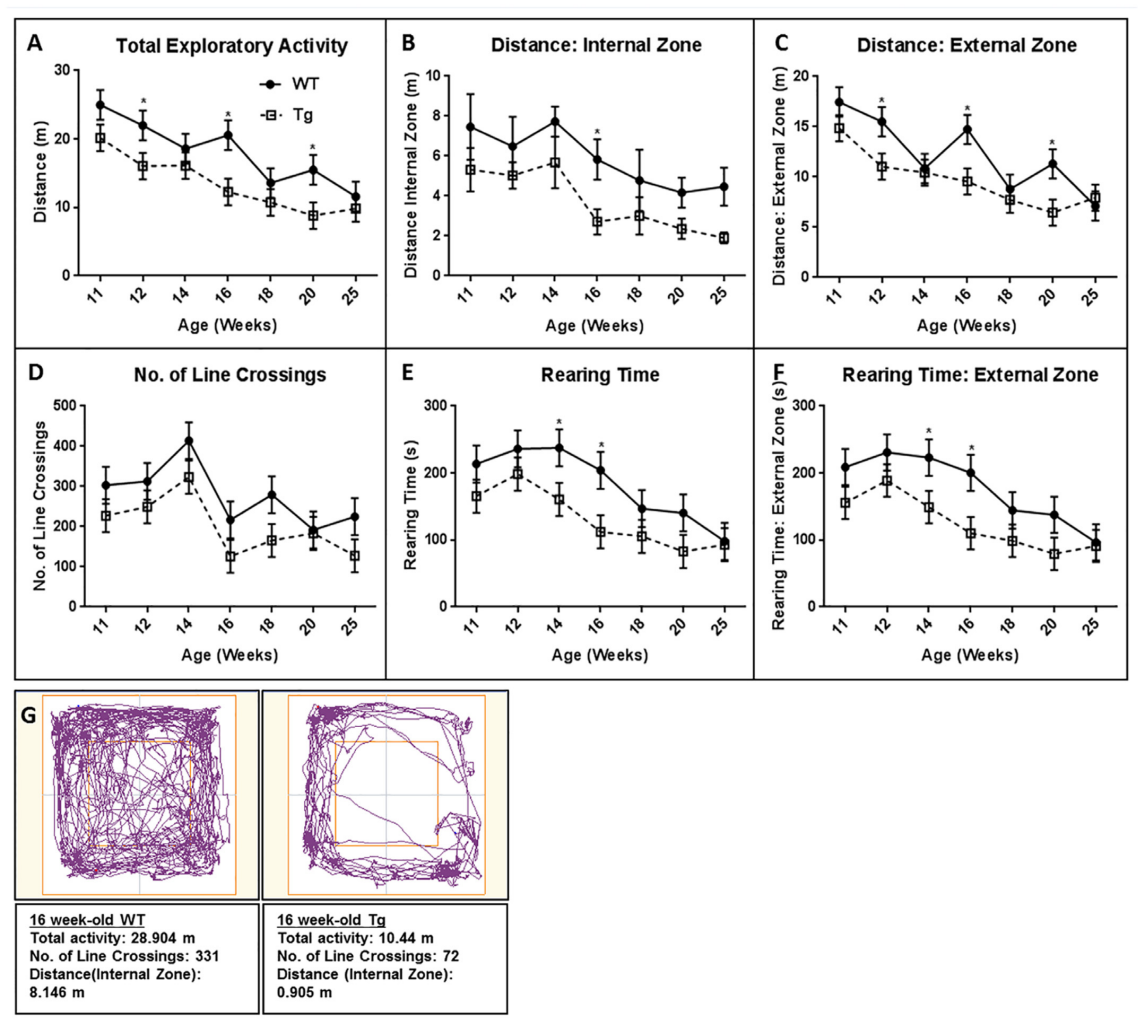
Longitudinal Open Field behavior comparison between HIV-1 Tg and age-matched WT rats. Open field measures were collected and plotted to the nearest whole age number (weeks): (A) Total Exploratory Activity (B) Total distance (Internal Zone) (C) Total distance (External zone) (D) Number of Line Crossings (E) Rearing time and (F) Rearing Time (External zone). The transgene state (group) and age were found to be significant predictors of performance [p<0.05], with the group and age interaction not statistically significant [p>0.05] except for total distance (External Zone). (G) Depicts representative track plots for 16 week-old WT and Tg rats. Each track represents the total activity, line crossings and distance traveled in the internal zone by the subject during the 30 min time period of the test. The WT track crosses into the center portion of the maze at regular intervals and exhibits high exploratory activity while the Tg rat remains in close proximity to the walls of the maze and shows decreased exploratory activity. Error bars reflect standard error of the mean (SEM) values. * reflect differences between WT and Tg rats (p<0.05).

On all activity measures (total exploratory activity, distance traveled (internal zone), distance traveled (external zone), number of line crossings, rearing time, and rearing time (external zone)), the WT rats generally outperformed the Tg rats (Fig 2A–2E). The transgene state (group) and age were found to be significant predictors of all these measures, while the group and age interaction was not significant for any measure except the distance traveled (external zone) (Table 1). When we looked at the percentage time spent in the external versus the internal zone, we found that the Tg rats had a greater percentage of their movements in the external zone compared to age-matched WT rats in the majority of the trials (5 out of 7 time points). This was most noticeable at 16 and at 25 weeks of age where the Tg rats spent 21% and 20% of their time in the internal zone respectively compared to 28% and 38% for the WT rats. An example of open field track plots from two 16 week-old Tg and WT rats are shown in Fig 2G: while the WT rat track crosses into the center portion of the maze at regular intervals, the Tg rat does not and remains in close proximity to the walls of the maze.

**Table 1:** Open Field activity F statistics.

**Total Exploratory Activity**		
Group (transgene status)	F(1,8.85) = 8.94	p = 0.0155
Age	F(6,36.7) = 6.45	p = 0.0001
Interaction	F(6,36.7) = 1.55	p = 0.1898
**Distance Traveled (internal zone)**		
Group (transgene status)	F(1, 10.2) = 6.98	p = 0.0243
Age	F(6, 38.9) = 3.52	p = 0.007
Interaction	F(6, 38.9) = 0.27	p = 0.9465
**Distance Traveled (external zone)**		
Group (transgene status)	F(1, 8.7) = 6.22	p = 0.0350
Age	F(6, 36) = 8.89	p<0.0001
Interaction	F(6,36) = 3.01	p = 0.0172
**Number of Line Crossings**		
Group (transgene status)	F(1, 16.7) = 9.64	p = 0.0065
Age	F(6, 38.1) = 5.32	p = 0.0005
Interaction	F(6, 38.1) = 0.34	p = 0.9120
**Rearing time**		
Group (transgene status)	F(1, 12.6) = 7.41	p = 0.0179
Age	F(6, 39.2) = 4.35	p = 0.0019
Interaction	F(6, 39.3) = 0.69	p = 0.6589
**Rearing time (external zone)**		
Group (transgene status)	F(1, 11.7) = 7.47	p = 0.0185
Age	F(6, 39.3) = 3.88	p = 0.0039
Interaction	F(6, 39.3) = 0.69	p = 0.6589

### ^18^F-FDG PET scanning

**Fig 3:**
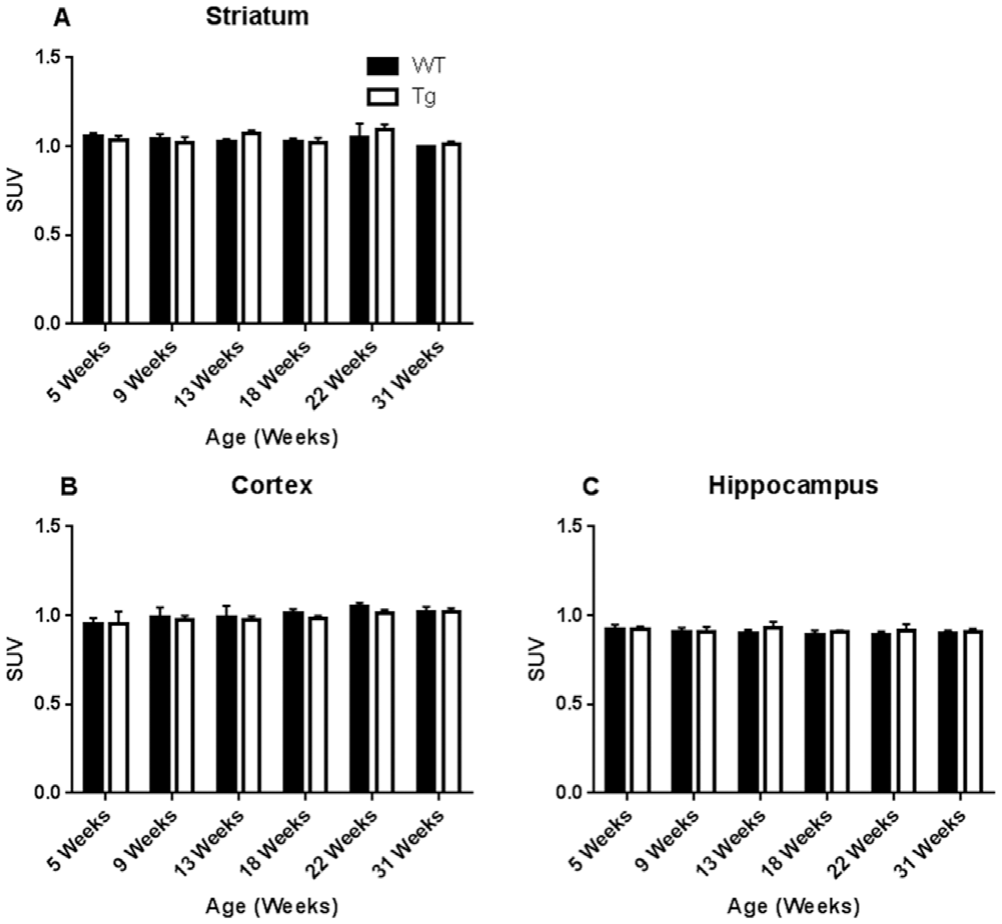
^18^F-FDG uptake analysis between Tg and WT rats by PET scan (longitudinal study group). Assessment of metabolic activity, determined by ^18^F-FDG uptake (SUV) normalized to whole brain uptake was performed for specific brain regions: (A) Striatum, (B) Cortex and (C) Hippocampus. Differences were not statistically significant at any time point (p>0.05; Student’s *t*-test). Error bars reflect standard deviation (SD) values.

**Fig 4:**
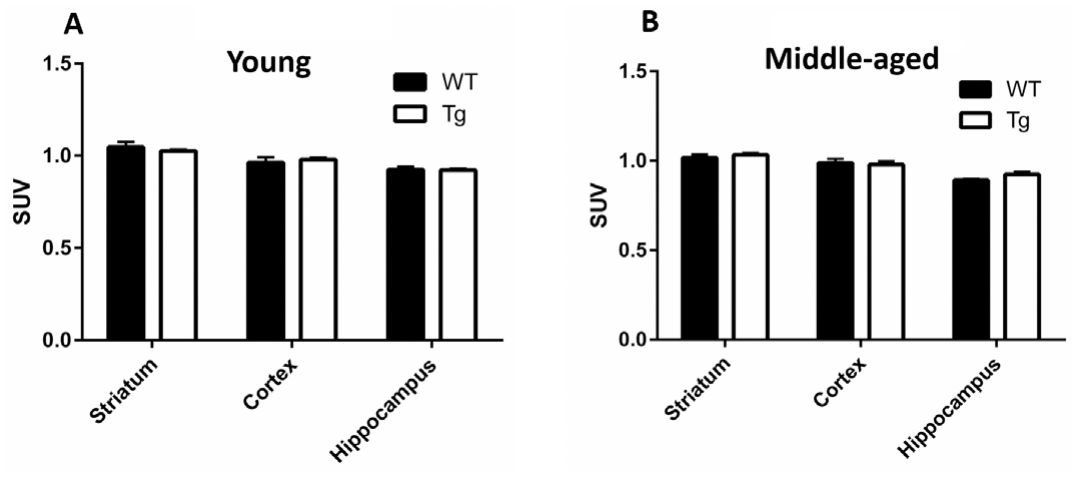
^18^F-FDG uptake analysis between Tg and WT rats by PET scan (cross-sectional study group). Assessment of metabolic activity in (A) young (12 week-old) rats and (B) middle-aged (29 week-old) rats, determined by ^18^F-FDG uptake (SUV) normalized to whole brain uptake was performed for Striatum, Cortex and Hippocampus. Differences were not statistically significant between the Tg and WT rats at either age (p>0.05; unpaired Student’s *t*-tests). Error bars reflect standard deviation (SD) values.

The same animals that underwent rotarod and open field behavioral testing underwent ^18^F-FDG -PET scans (total of 9 rats in two groups: 4 WT and 5 Tg). Longitudinal differences in ^18^F-FDG uptake (SUV corrected to whole brain uptake) in the same animals within the striatum, hippocampus and cortex (somatosensory and motor) by PET did not reach significance when baseline values (5 week-old) were compared to the last scan values (31 week-old) neither in the Tg nor in the WT rats (p>0.05, paired Student’s *t*-test). There were also no significant differences in uptake between Tg and WT rats at any specific time point (p>0.05, unpaired Student’s *t*-tests) (Fig 3A–3C). Furthermore, a separate cross-sectional examination of ^18^F-FDG uptake data between young Tg and WT rats (around 12 week-old) and between middle-aged Tg and WT rats (around 29 week-old) also did not identify significant differences in uptake (p>0.05, unpaired Student’s *t*-test) (Fig 4).

### ^14^C-DG Autoradiography

**Fig 5:**
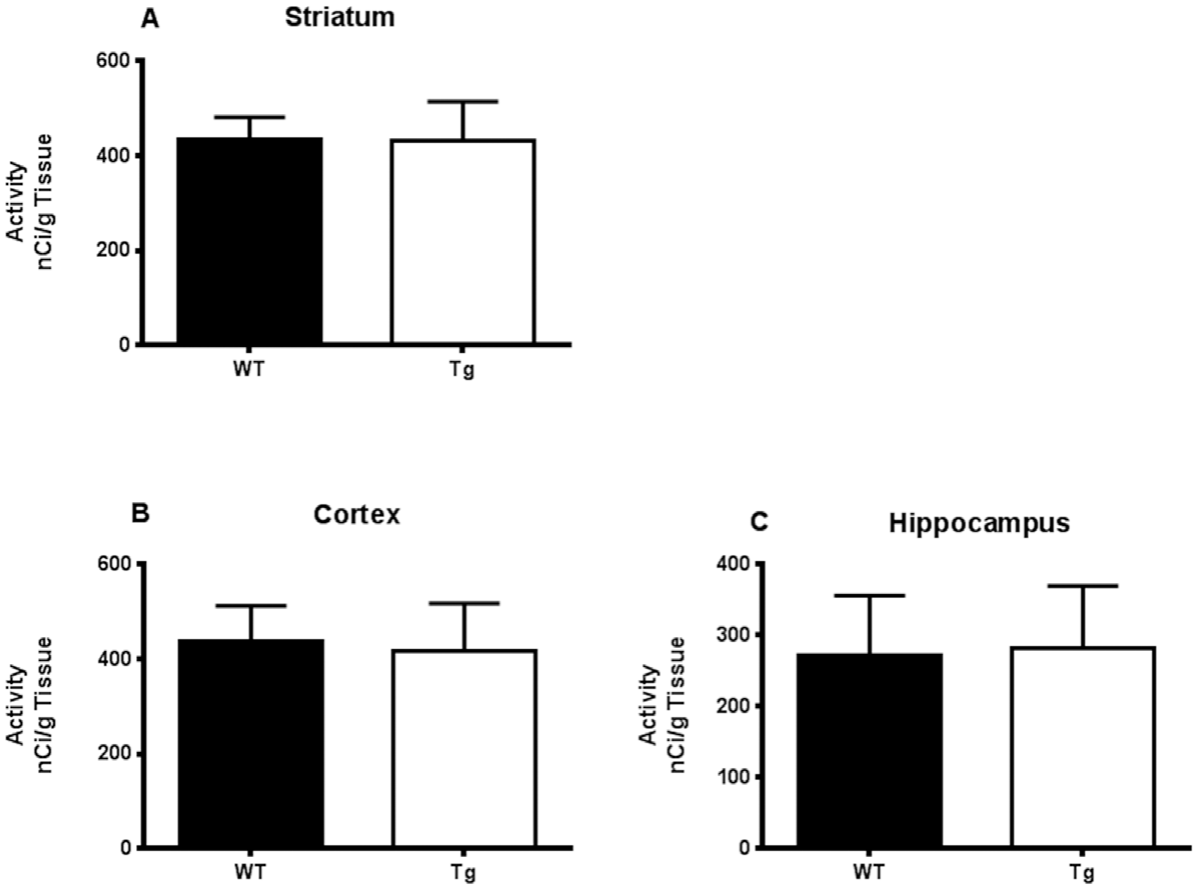
^14^C-DG uptake analysis between Tg and WT rats by autoradiography. A cross-sectional analysis of ^14^C-DG (nCi/g) uptake was performed on 8 month-old HIV-1 Tg and aged-matched WT rats for specific brain regions: (A) Striatum, (B) Cortex and (C) Hippocampus. Differences of radioactivity concentrations (nCi/g) were not statistically significant (p>0.05; Mann-Whitney U-test). Error bars reflect standard deviation (SD) values.

Consistent with ^18^F-FDG-PET results at 31 weeks (Fig 3A–3C), ^14^C-DG uptake values (in nCi/g) in the cortex, [439.32 ± 63.74; 418.12 ± 86.24], striatum [434.41 ± 40.96; 433.29 ± 70.42] and hippocampus [271.84 ± 72.91; 281.27 ± 76.29] brain sections were not significantly different (p>0.05; mean ± SD) at 32–33 weeks between WT and Tg rats (Fig 5A–5C).

## Discussion

The introduction of HAART has resulted in significant decrease in the morbidity, mortality, and neuropathology of HIV infected patients; however, HAND is still a common problem of infected patients [1–3] and subsequently neuroprotective therapies are being developed and preclinical testing is needed. Neuro-HIV research could thus benefit from well described small animal models used to test those neuroprotective therapies; among those models, the HIV-1 Tg rat is used by many groups, is commercially available, non-infectious and demonstrates many of the clinical features of patients with HAND [15, 18, 26, 27]. We have previously reported decreased total brain, parenchymal and striatal volumes, decreased striatal tyrosine hydroxylase (TH) antibody staining, decreased striatal dopamine D2/3 receptor binding, as well as increased corpus callosum mean diffusivity/decreased fractional anisotropy on diffusion tensor imaging (DTI) in the HIV-1 Tg rat brain compared to age-matched WT rats [28, 29]. At the same time we were not able to demonstrate measurable microglial activation by PET imaging [30]. Those findings corresponded to histopathological abnormalities suggesting neurodegeneration in the Tg rat. In our current study we wanted to see if these structural and functional changes translate into longitudinal differences in glucose metabolism as visualized by ^18^F-FDG-PET imaging, considering the wide availability of this ligand for imaging and the relative simplicity of the analysis. At the same time we wanted to document that the metabolic activity we are measuring in the brains of the HIV-1 Tg rats does correspond to motor and behavioral abnormalities.

Rotarod testing at constant speed is used to assess motor competence, coordination and balance [31]. With accelerating rod rotation rates however, performance depends more on motor planning rather than locomotor capabilities [15]. Rotarod testing of Tg rats has been performed previously in a cross-sectional setting in which the rats performed similarly on the non- accelerating rotarod, suggesting relatively preserved motor function in the Tg rats. However, when tested on the accelerating rotarod, Tg rats performed worse than controls, suggesting deficits in motor planning and/or learning [20]. In our study, we performed longitudinal assessment of motor performance and planning, using the accelerating model of rotarod. WT rats in our hands generally outperformed the Tg rats at most points, especially before habituation, reproducing longitudinally the previous cross–sectional work. However in our longitudinal setting, we were also able to assess habituation rates since the rats were evaluated at multiple points as they grew older. Habituation, a form of non-associative learning, is defined as a gradual decrease in behavioral response that results from repeated stimulation and does not involve sensory adaptation/sensory fatigue or motor fatigue [32, 33]. In our rats, we measured habituation as the earliest point where rotarod performance differed significantly from the baseline. As expected, even though habituation occurred in the WT rats at week 8 of age (fifth testing session), it never did in the Tg rats. Similar deficits of habituation to other motor/behavioral tests have been previously described in Tg rats: in running wheel behavioral testing of the Tg rats, they were found to persistently run more than the WT rats after five days of testing, indicating less habituation of the behavior [19].

We also evaluated the same group of animals for exploratory behavior, locomotor activity as well as anxiety-related behaviors using open field testing [34]. Reports have demonstrated that rats with increased emotionality show reduced total motor activity, reduced distance travelled in central zone and reduced vertical activity. Non-emotional animals, on the other hand, move throughout the novel environment, show high levels of vertical activity and respond to the open field with continuous activity [34–37]. In our longitudinal assessment of behavioral measures, the WT rats outperformed the Tg rats in all measures at almost all time points (Fig 2). The presence of the transgene was found to be a significant predictor of decreased responses to these measures in the Tg rat, suggesting higher emotionality and/or anxiety levels (Table 1 and Fig 2A–2F). Anxiety was best demonstrated in the Tg rats travelling more frequently in the external zone compared to the WT rats.

Despite the above described motor and behavioral deficits, the longitudinal and cross sectional differences in glucose metabolism as measured by ^18^F-FDG-PET did not reach significance between the HIV-1 Tg and WT rats (Figs 3 and 4). These data were further confirmed by the lack of significant difference in glucose metabolism as measured by ^14^C-DG-autoradiography (Fig 5). However, we have previously published imaging and histological data [28, 29] compatible with significant striatal pathology in the Tg rats compared to WT. The explanation for this discrepancy could lie in the nature of the measured parameters: in order to see decreased brain metabolic activity using ^18^F-FDG-PET, neurotoxicity resulting in neuronal degeneration and death should first occur [38] and this might not be happening in our animals to the point of being detected by ^18^F-FDG-PET. In fact, even though we have previously shown decreased NeuN counts in the older Tg animals compared to WT [39], those results were not statistically significant. This is in contradistinction to our previous findings using ^18^F-fallypride PET imaging and TH staining, showing significant striatal dopaminergic dysfunction in the Tg rat. So, even though the neurons in the Tg rat are not degenerating to the point of significantly losing their basic metabolic activity, they still are functionally deficient. Those functional deficits could explain, at least partially, the motor and behavioral abnormalities we saw in our animals and the deficits seen by other groups as well [11, 12, 15–18, 40].

^18^F-FDG-PET abnormalities in the brain of HIV-positive patients were significant in the pre-ART era [41–43] however became less dramatic in the post ART era [44, 45]. This probably reflects a more subtle pattern of neurotoxicity in optimally treated HIV patients, which does not directly kill the neurons as in the pre ART era. We have shown subtle pathology in our Tg animals, with behavioral and motor deficits, but no overt neuronal death, similar to neuropsychiatric deficits in treated HIV-positive patients with no overt cellular loss or productive infection. Those findings further argue to the potential value of the Tg rat as a model of treated HIV-positive patients.

## Conclusion

In conclusion, even though ^18^F-FDG uptake by PET can detect differences in glucose metabolism and is a practical bio-marker for neurodegeneration, it does not appear to be sensitive enough to be used as a biomarker of neuropathology in the HIV-1Tg rat model. More specific biomarkers, such as those targeting various neurotransmitter systems, or targeting oxidative stress markers are thus needed in this animal model.
